# Predictive Values of Inflammation-Related Markers and Thyroid Function in Pediatric Thyroid Cancer Patients

**DOI:** 10.3389/fped.2021.802214

**Published:** 2021-12-24

**Authors:** Shuo Li, Yihao Liu, Shaoxuan Liu, Gongbo Du, Zipeng Wang, Detao Yin

**Affiliations:** ^1^Department of Thyroid Surgery, The First Affiliated Hospital of Zhengzhou University, Zhengzhou, China; ^2^Department of Anethesiology, The First Affiliated Hospital of Zhengzhou University, Zhengzhou, China; ^3^Key Discipline Laboratory of Clinical Medicine Henan, Zhengzhou, China

**Keywords:** inflammation-related markers, neutrophil-lymphocyte ratio, thyroid function, TSH, pediatric thyroid cancer

## Abstract

Few researchers have studied the diagnostic value of inflammation-related hematological indexes of pediatric thyroid carcinoma exclusively. Whether thyroid-stimulating hormone (TSH) is an independent risk factor for pediatric thyroid cancer is still controversial. To assess the correlativity and predictive values of inflammation-related markers and thyroid function in pediatric thyroid cancer patients, we collected a total of 270 children with thyroid nodules for two consecutive years. Clinical data including age, gender, thyroid function, inflammation indexes, and clinical pathologic finding were collected and analyzed. The above-mentioned data were compared between the benign group and the malignant group, followed by the subgroups comparison. Binary logistic regression analysis was used to evaluate the correlation of markers and the pathological features of thyroid nodules. The neutrophil-to-lymphocyte ratio (NLR) showed a significant difference between thyroid cancer and thyroid nodules, while TSH did not. NLR > 1.49529 was the prognostic indicator of pediatric thyroid cancer. The logistic regression model further revealed that NLR > 1.49529 was an independent risk factor for thyroid cancer in pediatric patients. Furthermore, TSH was not correlated with the tumor characteristics in the thyroid cancer group. In conclusion, the findings in this study showed that NLR could be a predictor of thyroid cancer in pediatric patients and refuted the present view that TSH is a risk factor in pediatric thyroid cancer.

## Introduction

Thyroid cancer (TC) is a major component of pediatric endocrine tumors. TC accounts for more than 6% of children with cancer, and in the past 40 years, this number has been on the rise (1). Compared to adults, thyroid nodules (TN) have a lower incidence in pediatric patients. However, the malignancy rate is 1/4, which is significantly higher than that in adults (2). Papillary thyroid carcinoma (PTC) is the most common subtype of TC, followed by follicular thyroid carcinoma (FTC) (3). In the past decades, the diagnosis and treatment guideline for pediatric TC is largely inferred from that of guidelines for adults. However, pediatric TC is different from the adult type in many ways, including clinical symptoms, pathophysiology, and long-term results (2). Thus, it is important to study pediatric TC separately.

An increased number of studies reported that cancer development is remarkably correlated with the tumor microenvironment and inflammatory responses. Inflammation-related markers, including the neutrophil-to-lymphocyte ratio (NLR), the lymphocyte-to-monocyte ratio (LMR), the platelet-to-lymphocyte ratio (PLR), mean platelet volume (MPV), and platelet distribution width (PDW), have become new study hotspots (4). A systematic review and meta-analysis indicated that the NLR was associated with the balance of the immune system and the survival of solid tumors (5). CD4 and CD8 T cells, as well as a variety of cytokines and chemokines, regulate anti-tumor immune responses. The NLR reflects the level of cytokines and chemokines (6). In previous studies, an increasing NLR was associated with a high risk of recurrence and unfavorable prognosis of PTC (7). Moreover, a high NLR was also associated with extrathyroidal invasion, bilateral multiple focal, and tumor lymph node-positive of PTC (8). However, only a few studies reported on the relationship between NLR and pediatric TC.

Multiple studies reported that elevated thyroid-stimulating hormone (TSH) is a risk factor in TC and that TSH can be used as an auxiliary diagnosis, in which the mechanism may involve the proliferative features of TSH (9, 10). However, relevant studies on pediatric patients still have apparent defects and remain unclear. For example, in several studies on pediatric TC, thyroiditis and nodule characteristics were not considered (11). Autoimmune thyroiditis, coexisting with TC (12), is reported in lots of papers and is also found with elevated TSH (13). Whether serum TSH can be used as an independent risk factor for pediatric TC remains to be explored.

In this study, we aimed to assess the risk factors in pediatric TC and identify the potential prognostic indicator in blood.

## Materials and Methods

### Patients

This study retrospectively collected data from Chinese children with TN in the First Affiliated Hospital of Zhengzhou University for three consecutive years (from January 2018 to December 2020). The patient's clinical, laboratory, and imaging findings and cytological and pathological data were collected through the hospital registry system. The study was approved by the First Affiliated Hospital of Zhengzhou University ethics committee.

### Inclusion and Exclusion Criteria

We collected data on pediatric patients (age <18 years) with TN diagnosed by thyroid ultrasound (US). Inclusion criteria were as follows: (1) at least one TN identified by the US and (2) the result of blood routine examination, serum TSH, and thyroid auto-antibodies measured 7 days before or after thyroid US (before thyroid surgery, if it was performed). Exclusion criteria were as follows: (1) patients with previous cases of thyroid surgery; (2) patients with Graves disease; (3) patients with diabetes; (4) patients with pituitary dysfunction; (5) patients with benign or malignant non-thyroid tumor; (6) patients with degree 4 and above thyroid US but without pathological result; and (7) patients with an immunocompromised condition.

### Group

In total, 270 patients (184 girls, 86 boys) were included in this study. According to the clinical and pathological data, patients were divided into two groups: patients with malignant pathological or cytological evidence were considered as the TC group (*n* = 89); the remainder of the patients were considered the benign TN group (*n* = 181), All patients in TC group received thyroidectomy, including 54 patients for bilateral thyroid lobectomy and 35 patients for unilateral thyroidectomy. Patients in the TN group did not undergo thyroidectomy for at least 12 months of follow-up. Furthermore, each patient was recommended to have thyroid US performed every 3–6 months to confirm the nature of the benign nodules.

### Statistical Analysis

We retrospectively collected data from children with TN, including age, gender, thyroid function, and clinical pathologic finding. Thyroid function is reflected in free thyroid hormones (FT_3_, FT_4_), anti-thyroid peroxidase antibodies (TPOAb), and anti-thyroglobulin antibodies (TgAb). Data regarding the inflammation indexes, including complete neutrophils count, blood platelet count, and lymphocytes count of the preoperative routine blood test were also collected. Next, we calculated the key values like NLR (divide lymphocytes count into neutrophils count), LMR (divide monocyte count into lymphocytes count), and PLR (divide lymphocytes count into blood platelet count) separately.

All statistical analyses were performed using the SPSS 21.0 software (IB, USA). Continuous variables are expressed as the mean ± standard deviation (SD), and classification variables are presented as percentages. Values without normal distribution are expressed as the median and interquartile range (25–75%). For comparison, the *t*-test was performed for normally distributed data, while the Mann–Whitney U-test was performed for non-normally distributed data. The chi-square test was used for classified variables. The receiver operating characteristics (ROC) curve, based on the evaluation of the area under the curve (AUC) and 95% confidence interval (CI), was performed to identify the optimal cut-off value for blood parameters. The univariate and multivariate analyses were conducted to assess prognostic factors with logistic regression models. *p* < 0.05 was thought to have a statistically significant difference; the *p*-value is based on the bidirectional test.

## Results

### Description of the Patients' Baseline Characteristics

Table 1 shows the baseline characteristics of the 270 pediatric patients with TN. The median age was 15 years (ranging from 2 to 17 years), 86 patients (31.85%) were boys, and 96 patients (35.56%) were <14 years old. Between the benign nodule group and the TC group, there was a significant difference in sex (*p* = 0.004), but there was no significant difference in age (~14 and 14–17 years old, *p* = 0.324).

**Table 1:** Demographic and clinical characteristics of pediatric thyroid cancer patients.

	**Total**	**Thyroid cancer**	**Thyroid nodule**	***p***
Total	270 (100%)	89 (33%)	181 (67%)	
Age (years)				
<14	96 (35.6%)	28 (31.5%)	68 (37.6%)	0.324
<17	174 (64.4%)	61 (68.5%)	113 (62.4%)	
Gender				
Boys	86 (31.9%)	18 (20.2%)	68 (37.6%)	0.004
Girls	184 (68.1%)	71 (79.8%)	113 (62.4%)	
Hypoechogenicity				
Yes	80 (29.6%)	62 (69.7%)	18 (9.9%)	0
No	190 (70.4%)	27 (30.3%)	163 (90.1%)	
FT3 (pmol/L)	5.5	5.5	5.505	0.559
FT4 (pmol/L)	11.18	10.99	11.32	0.121
TSH (μIU/ML)	2.44	2.8	2.37	0.158
TPOAb(+)	36	18	18	0.012
TgAb(+)	46	28	18	0.000
LMR	5.452	5.23	5.5	0.112
NLR	1.344	2.19	1.3	0.004
PLR	115.185	116.8	114.29	0.881

*Data are expressed as median*.

*The significance level was set at p < 0.05*.

### Difference in Clinical Findings Between TC and TN

Clinical data of the benign and malignant pediatric TN patients are presented in Table 1. We found significant differences between the TN group and the TC group in hypoechogenicity of nodules (*p* = 0), NLR (*p* = 0.004), the TPOAb positive rate (*p* = 0.012) and the TgAb positive rate (*p* = 0). There were no significant differences in FT_3_, FT_4_, TSH, LMR, and PLR between the two groups.

For further research, we analyzed the above-mentioned clinical data by subgroup analysis, based on the patients' age and sex. Results are shown in Supplementary Table 1. As for patients younger than 14 years of age, the only significant difference found between TN and TC was TgAb (*p* = 0.004). Moreover, in the elder crowd (14–18 years), NLR (*p* = 0.01) and TgAb (*p* = 0.001) were different between the two groups. Also, we conducted a similar analysis for boys and girls. In boys, a significant difference in clinical data was observed for LMR (*p* = 0.005), while in girls significant differences were observed for NLR (*p* = 0.024), TPOAb (*p* = 0.041), and TgAb (*p* = 0).

**Figure 1:**
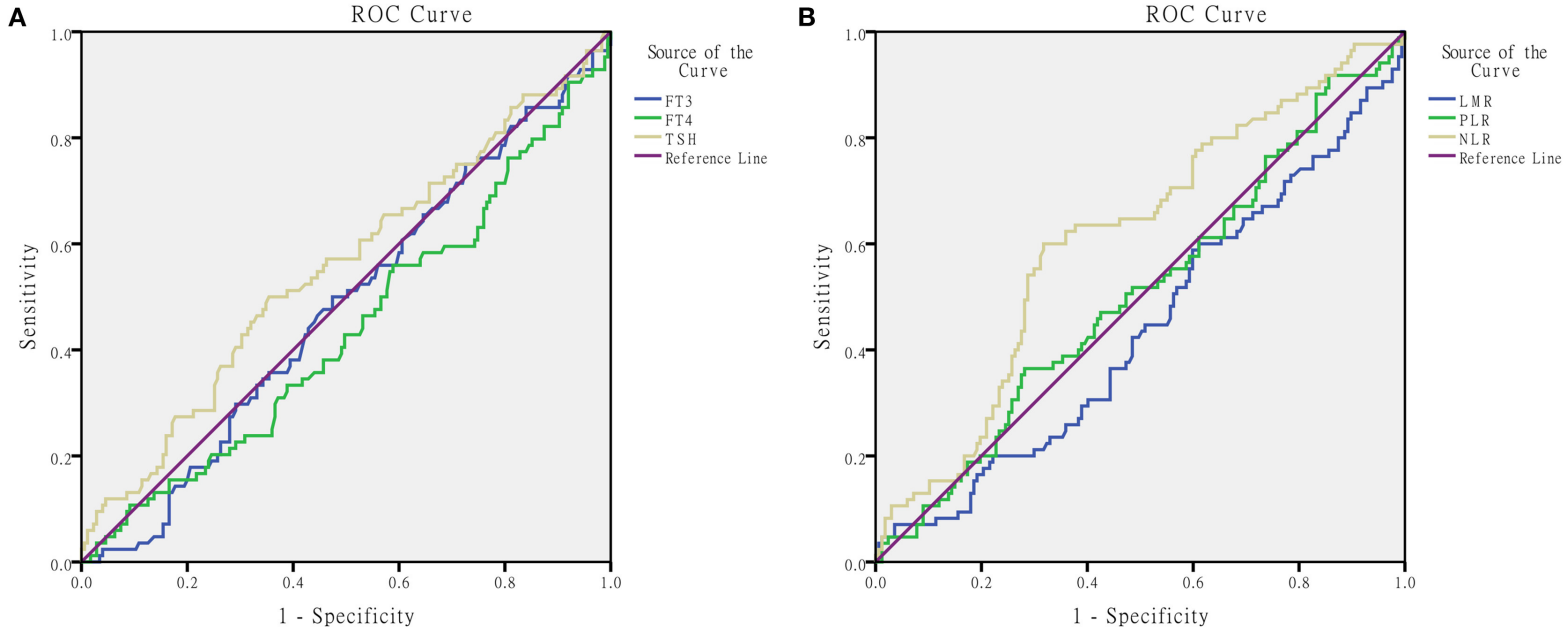
ROC for determination of predictive ability of thyroid function **(A)** and peripheral blood inflammatory indicators **(B)** in pediatric thyroid cancer patients.

ROC curve analyses were performed to predict FT_3_, FT_4_, TSH, LMR, NLR, and PLR, respectively. Figure 1, Table 2 show the results. The AUC value of NLR for predicting bilaterality was 0.610 (95% CI 0.637–0.684; *p* = 0.004). The best cut-off value was 1.49529, with a sensitivity of 60% and a specificity of 68.3%. The AUC for LMR, PLR, FT_3_, FT_4_, and TSH for prediction were 0.439 (*p* = 0.112), 0.506 (*p* = 0.881), 0.48 (*p* = 0.609), 0.439 (*p* = 0.11), and 0.554 (*p* = 0.158), respectively.

**Table 2:** AUC of peripheral blood inflammatory indicators in pediatric patients with thyroid cancer.

	**AUC**	***p***	**Cut-off value**
LMR	0.439	0.112	
PLR	0.506	0.881	
NLR	0.61	0.004	1.49529
FT3	0.48	0.609	
FT4	0.439	0.110	
TSH	0.554	0.158	

*The significance level was set at p < 0.05*.

Group-based analyses are shown in Supplementary Figures 1, 2, and Supplementary Table 2. For patients younger than 14 years of age, there was no predictive value for each indicator: FT_3_, FT_4_, TSH, LMR, NLR, and PLR, while in the age group 14–17 years, the AUC of NLR was 0.623 (95% CI 0.536–0.711; *p* = 0.01). The best cut-off value was 1.502, with a sensitivity of 63.2% and a specificity of 65.4%. The AUC for NLR in the girls group was 0.602 (95% CI 0.516–0.689; *p* = 0.024). The best cut-off value was 1.5, with a sensitivity of 58.2% and a specificity of 67.9%.

### Correlations Between Inflammation-Related Markers and Pediatric Thyroid Cancer

**Figure 2:**
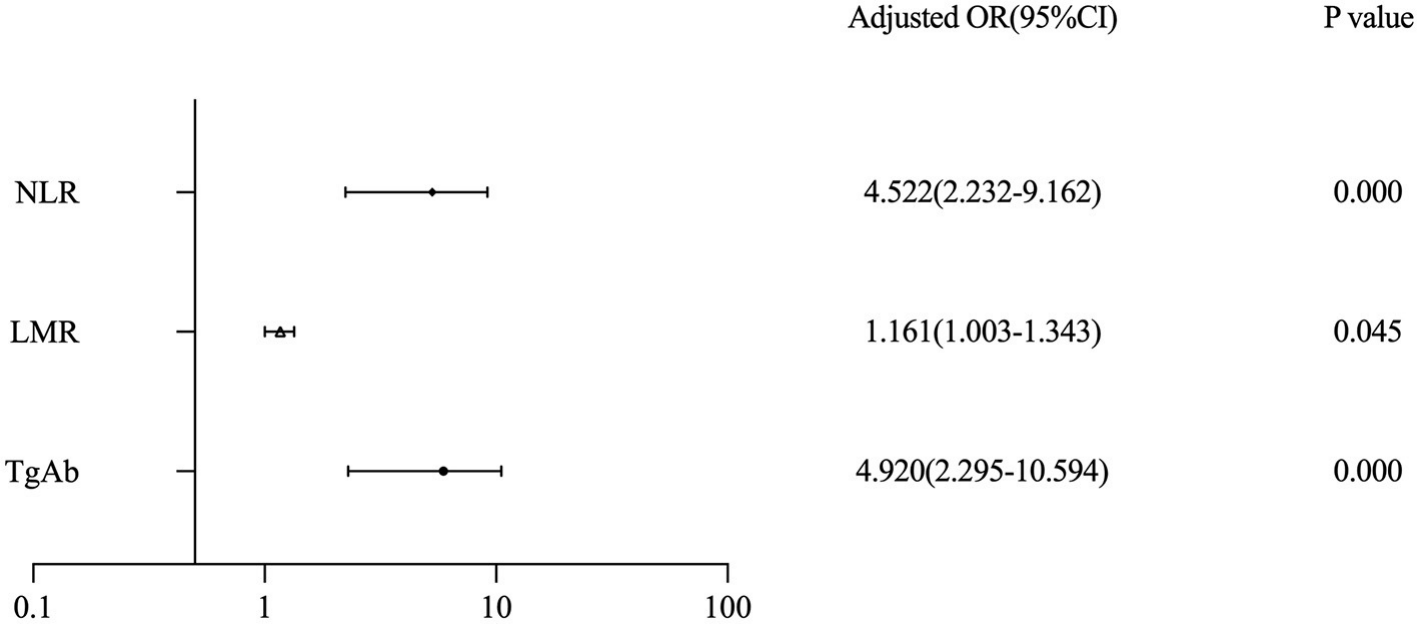
Forest plot of three datasets in pediatric patients with thyroid cancer.

Univariate and multivariate logistic analyses were conducted to further identify whether the lymphocyte count and thyroid function values were independently correlated to pediatric TC. The results are presented in Table 3. Using the NLR cut-off points calculated by ROC curve analysis, the results showed that NLR > 1.49529 was an independent risk factor (OR = 3.226, *p* = 0) for pediatric TC. Moreover, multivariate analyses showed in Figure 2 that NLR > 1.49529 was associated with TC (*p* = 0, OR = 4.522), as were TgAb (*p* = 0, OR = 4.92) and LMR (*p* = 0.045, OR = 1.161).

**Table 3:** Univariate and multivariate analysis for pediatric thyroid cancer.

	**Univariate analysis**		**Multivariate analysis**	
	**OR (95% CI)**	***p***	**OR (95% CI)**	***p***
Age	1.059	0.232		
Gender	2.374	0.005		
TPOAb(+)	2.446	0.014		
TgAb(+)	0.444	0.000	4.92	0.000
FT3	0.84	0.16		
FT4	0.91	0.092		
TSH	1.055	0.178		
LMR	0.946	0.362	1.161	0.045
PLR	1	0.906		
NLR	3.226	0.000	4.522	0.000

*The significance level was set at p < 0.05*.

## Discussion

TC is a major component of pediatric endocrine cancer, and its proportion has been increasing in the past four decades (1). In the past decades, there is no diagnosis and treatment guideline for pediatric TC; therefore, the thyroid surgeon could only follow the adult guidelines. In 2015, the American Thyroid Association (ATA) published guidelines for pediatric differentiated TC patients for the first time (2). The guidelines showed that pediatric TC was different from adult type in many ways, including clinical symptoms, pathophysiology, and long-term results. Compared to adult TN, it has a lower incidence in pediatric patients. However, the malignancy rate is 1/4, which is significantly higher than that in adults (2). The main bias of guidelines is the lack of detailed data and research, despite the particularity of the treatment and the fact that prognosis of pediatric differentiated TC patients have been clarified (14). Hence, further studies on pediatric TC remain to be investigated.

For pediatric patients with TN, there are two common methods for diagnosis: thyroid US and fine-needle aspiration (FNA) biopsy (2). The US is used to determine which nodule(s) should be further evaluated. The next step after the US is to perform an FNA biopsy to obtain cells from the nodule and investigate them microscopically. Thyroid US for health examination in children is not as widely used as in the middle-aged and elderly population. Thus, many pediatric TC patients cannot be detected and treated as early as possible. Moreover, FNA biopsy has limitations in clinical use, because it involves an invasive procedure. Therefore, identifying a non-invasive, low-cost, and easily available modality is of utmost importance.

In previous studies, the relationship between the tumor and chronic inflammation has been well studied (15). Neutrophils have been reported to promote tumor progression, while lymphocytes suppressed tumor progression (16). Wen et al. showed that the increase in LMR independently contributed to an advanced TNM stage in PTC patients ≥55 years, while the NLR was not associated with nodule features and the TNM stage in TC patients. Few studies have studied the relationship between the blood index and pediatric TC, and the diagnostic value of the hematological index is still questionable (17).

The aim of this study was to study the possible clinical variables and investigate the reliability of inflammation-related hematological indexes to predict disease persistence in pediatric patients with TC.

Our study firstly suggested that preoperative NLR was positively associated with pediatric TC; we considered NLR as a predictive indicator. According to the result of baseline characteristic comparison, there was a significant difference between boys and girls; only the group-based analysis was rigorous. Moreover, pediatric blood test results are influenced by age; therefore, statistical analyses were performed on both gender and age groups. We found a statistical difference in NLR between the TC group and the TN group, which was also found in the 14–17 year old group and the girls group, rather than in the ~14 year old group or the boys group. These results can be explained by the fact that TC group and TN group were different in gender composition (*p* = 0.004); there were more female patients than male patients. Another explanation is that the sample size in the boys group (*n* = 86) and ~14 year old group (*n* = 96) were too small to reveal the expected result.

To explore predictive values, we draw the ROC curve and calculated the best cut-off value of NLR as 1.49529 (*p* = 0.004). ROC with statistical significance was presented in 14–17 year old group and girls group, similar to the difference comparison. The above evidence showed that NLR could be considered as an auxiliary diagnosis indicator. Regression analysis was conducted and NLR > 1.49529 was the independent risk factor for pediatric TC in every method of regression analysis: univariate or multivariate, enter method or forward LR.

Neutrophils, as a part of the host immune reaction, exhibit different functions to dynamically regulate cancer-related processes. It has been proven that neutrophils induced by the tumor can promote tumor metastasis through circulation (18). Many clinical retrospective investigations verified the above-mentioned mechanism (19). Lymphocytes, basic players in the acquired immune system, are characterized by suppressing tumor cells proliferation, inhibiting migration, and destroying metastases (20). In addition, previous studies have confirmed that an increase in lymphocyte count had a positive effect on a better survival period of terminal cancer patients (21).

Multiple studies pointed that the elevated TSH was a risk factor in pediatric and adult TC, but some of these studies still had apparent defects and remained unclear. TSH was proposed as an auxiliary diagnosis indicator for TC (9, 10). However, this viewpoint is in contradiction with the findings presented by Holm et al. (21). In the study from Holm, 829 patients with hypothyroidism were enrolled, and no increased risk in TC was observed after 22 years of follow-up. Thus, it remains controversial whether TSH can be recognized as an independent risk factor to thyroid carcinogenesis.

Our findings demonstrated that serum TSH was not a risk factor in pediatric TN. The data showed that there was no statistically significant difference in TSH concentration between TN and TC (*p* = 0.158) and that there was a negative correlation with the malignancy of TN, which was consistent with the findings presented by Wang et al. (22). The possible underlying mechanism may be that the TSH receptor signal pathway was not sufficient to induce carcinogenesis (23).

Due to the inherent quality of cross-sectional studies, our study has some limitations. First, not all subjects in the benign nodules group had pathological or cytological evidence. Although we did at least 12 months of follow-up, there was still uncertainty. Secondly, all cases who suffered from the disease that could influence leucocytes or thyroid function were excluded, but the result may be influenced by other unknown or undetected factors. Finally, the clinical indicators that we collected were not changeless; they floated in some time. Our single measurement is insufficient. Furthermore, multicenter, prospective, or randomized controlled studies are needed to validate our conclusion.

In short, our retrospective study described inflammatory indexes in pediatric patients who have PTC firstly. We showed that the pretreatment peripheral index can be used as a cheap and convenient indicator of pediatric TC patients, including NLR and TgAb. It is necessary to pay more attention to the cut-off value NLR > 1.49529; the increase in NLR leads to the uprise of TC risk. Serum T_3_, T_4_, and TSH concentration does not seem to be a carcinogenic factor in pediatric patients with TN and is also not an independent risk factor in existing characteristics of thyroid carcinoma.

## Data Availability Statement

The raw data supporting the conclusions of this article will be made available by the authors, without undue reservation.

## Author Contributions

DY conceived of the idea and provided guidance. SLi wrote the manuscript and completed the figures. YL, SLiu, and ZW carefully reviewed the manuscript. GD made critical revisions to the manuscript. All authors contributed to the article and approved the submitted version.

## Funding

This study was funded by The University Scientific and Technological Innovation Team Project of Henan Province (19IRTSTHN002) and Major Scientific Research Projects of Traditional Chinese Medicine in Henan Province (No. 20-21ZYZD14).

## Conflict of Interest

The authors declare that the research was conducted in the absence of any commercial or financial relationships that could be construed as a potential conflict of interest.

## Publisher's Note

All claims expressed in this article are solely those of the authors and do not necessarily represent those of their affiliated organizations, or those of the publisher, the editors and the reviewers. Any product that may be evaluated in this article, or claim that may be made by its manufacturer, is not guaranteed or endorsed by the publisher.

## References

[1] Howlader N Noone AM Krapcho M Miller D Brest A Yu M. editors. SEER Cancer Statistics Review, 1975–2016. Bethesda, MD: National Cancer Institute (2019).

[2] Francis GL Waguespack SG Bauer AJ Angelos P Benvenga S Cerutti JM. Management guidelines for children with thyroid nodules and differentiated thyroid cancer. Thyroid. (2015) 25:716–59. 10.1089/thy.2014.0460 25900731 PMC4854274

[3] Bernier MO Withrow DR Berrington de Gonzalez A Lam CJK Linet MS Kitahara CM. Trends in pediatric thyroid cancer incidence in the United States, 1998-2013. Cancer. (2019) 125:2497–505. 10.1002/cncr.32125 31012956 PMC6602875

[4] Grivennikov SI Greten FR Karin M. Immunity, inflammation, and cancer. Cell. (2010) 140:883–99. 10.1016/j.cell.2010.01.025 20303878 PMC2866629

[5] Templeton AJ McNamara MG Šeruga B Vera-Badillo FE Aneja P Ocaña A. Prognostic role of neutrophil-to-lymphocyte ratio in solid tumors: a systematic review and meta-analysis. J Natl Cancer Inst. (2014) 106:dju124. 10.1093/jnci/dju124 24875653

[6] Chen ZY Raghav K Lieu CH Jiang ZQ Eng C Vauthey JN. Cytokine profile and prognostic significance of high neutrophil-lymphocyte ratio in colorectal cancer. Br J Cancer. (2015) 112:1088–97. 10.1038/bjc.2015.61 25688736 PMC4366901

[7] Kim JY Park T Jeong SH Jeong CY Ju YT Lee YJ. Prognostic importance of baseline neutrophil to lymphocyte ratio in patients with advanced papillary thyroid carcinomas. Endocrine. (2014) 46:526–31. 10.1007/s12020-013-0089-6 24272600

[8] Manatakis DK Tseleni-Balafouta S Balalis D Soulou VN Korkolis DP Sakorafas GH. Association of baseline neutrophil-to-lymphocyte ratio with clinicopathological characteristics of papillary thyroid carcinoma. Int J Endocrinol. (2017) 2017:8471235. 10.1155/2017/8471235 28572821 PMC5441114

[9] Zafón C Obiols G Mesa J. Preoperative TSH level and risk of thyroid cancer in patients with nodular thyroid disease: nodule size contribution. Endocrinol Nutr. (2015) 62:24–8. 10.1016/j.endonu.2014.06.002 25066642

[10] Shi RL Liao T Qu N Liang F Chen JY Ji QH. The usefulness of preoperative thyroid-stimulating hormone for predicting differentiated thyroid microcarcinoma. Otolaryngol Head Neck Surg. (2016) 154:256–62. 10.1177/0194599815618388 26598500

[11] Keskin M Savas-Erdeve S Aycan Z. Co-Existence of thyroid nodule and thyroid cancer in children and adolescents with hashimoto thyroiditis: a single-center study. Horm Res Paediatr. (2016) 85:181–7. 10.1159/000443143 26910846

[12] Won JH Lee JY Hong HS Jeong SH. Thyroid nodules and cancer in children and adolescents affected by Hashimoto's thyroiditis. Br J Radiol. (2018) 91:20180014. 10.1259/bjr.20180014 29595320 PMC6221765

[13] Ren PY Liu J Xue S Chen G. Pediatric differentiated thyroid carcinoma: the clinicopathological features and the coexistence of Hashimoto's thyroiditis. Asian J Surg. (2019) 42:112–9. 10.1016/j.asjsur.2017.10.006 29254871

[14] Cistaro A Quartuccio N Garganese MC Villani MF Altini C Pizzoferro M. Prognostic factors in children and adolescents with differentiated thyroid carcinoma treated with total thyroidectomy and RAI: a real-life multicentric study. Eur J Nucl Med Mol Imaging. (2021). 10.1007/s00259-021-05586-8. [Epub ahead of print]. 34664092 PMC8921094

[15] Elinav E Nowarski R Thaiss CA Hu B Jin C Flavell RA. Inflammation-induced cancer: crosstalk between tumours, immune cells and microorganisms. Nat Rev Cancer. (2013) 13:759–71. 10.1038/nrc3611 24154716

[16] Rosenberg SA: Progress in human tumour immunology and immunotherapy. Nature. 411:380–4. 10.1038/35077246 11357146

[17] Lang BH Ng CP Au KB Wong KP Wong KK Wan KY. Does preoperative neutrophil lymphocyte ratio predict risk of recurrence and occult central nodal metastasis in papillary thyroid carcinoma? World J Surg. (2014) 38:2605–12. 10.1007/s00268-014-2630-z 24809487

[18] Coffelt SB Wellenstein MD de Visser KE. Neutrophils in cancer: neutral no more. Nat Rev Cancer. (2016) 16:431–46. 10.1038/nrc.2016.52 27282249

[19] He JR Shen GP Ren ZF Qin H Gui C Zhang Y. Pretreatment levels of peripheral neutrophils and lymphocytes as independent prognostic factors in patients with nasopharyngeal carcinoma. Head Neck. (2012) 34:1769–76. 10.1002/hed.22008 22318781

[20] Bastid J Bonnefoy N Eliaou JF Bensussan A. Lymphocyte-derived interleukin-17A adds another brick in the wall of inflammation-induced breast carcinogenesis. Oncoimmunology. (2014) 3:e28273. 10.4161/onci.28273 25050201 PMC4063083

[21] Holm LE Blomgren H Löwhagen T. Cancer risks in patients with chronic lymphocytic thyroiditis. N Engl J Med. (1985) 312:601–4. 10.1056/NEJM198503073121001 3838363

[22] Wang G Ren N Wang S Zhang X Li Y Sun N. Serum TSH is not a risk factor for malignancy of pediatric thyroid nodules. Endocr Relat Cancer. (2021) 28:247–55. 10.1530/ERC-20-0508 33690161

[23] Franco AT Malaguarnera R Refetoff S Liao XH Lundsmith E Kimura S. Thyrotrophin receptor signaling dependence of Braf-induced thyroid tumor initiation in mice. Proc Natl Acad Sci U S A. (2011) 108:1615–20. 10.1073/pnas.1015557108 21220306 PMC3029699

