# Supplementary material for: Predictive Values of Inflammation-Related Markers and Thyroid Function in Pediatric Thyroid Cancer Patients
Source: Front Pediatr. 2021 Dec 24;9:802214. doi: 10.3389/fped.2021.802214 (PMC8740167; doi:10.3389/fped.2021.802214)
Supplement: Supplementary file 1 [file Data_Sheet_1.docx]

Supplementary Material

# Supplementary Figures and Tables

## Supplementary Figures


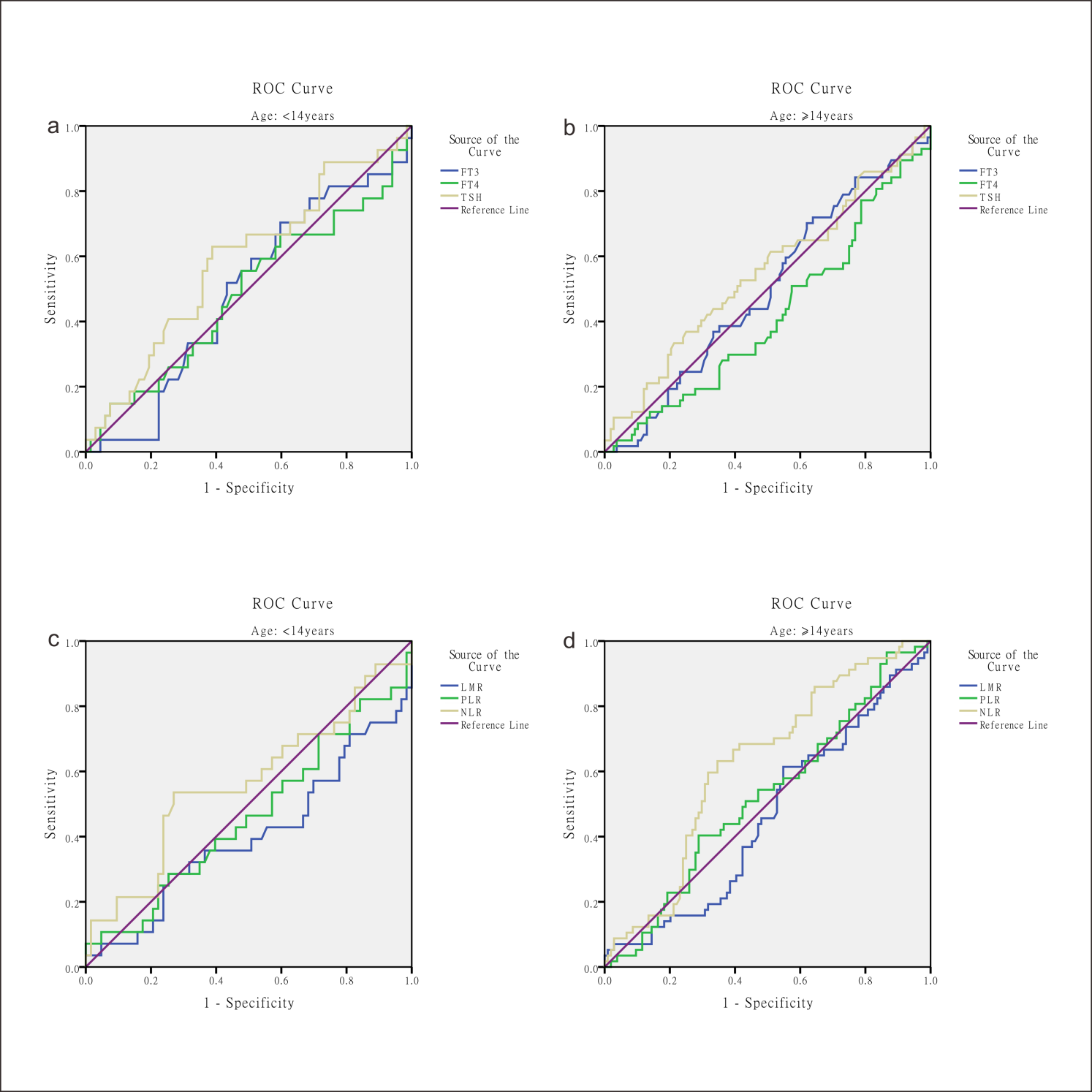


**Supplementary Figure 1.** ROC for determination of predictive ability of thyroid function and peripheral blood inflammatory indicators in pediatric thyroid cancer :a, thyroid function in ~14 years group. b, thyroid function in 14~17 years group. c, peripheral blood inflammatory indicators in ~14 years group. d, peripheral blood inflammatory indicators in 14~17years group.

**
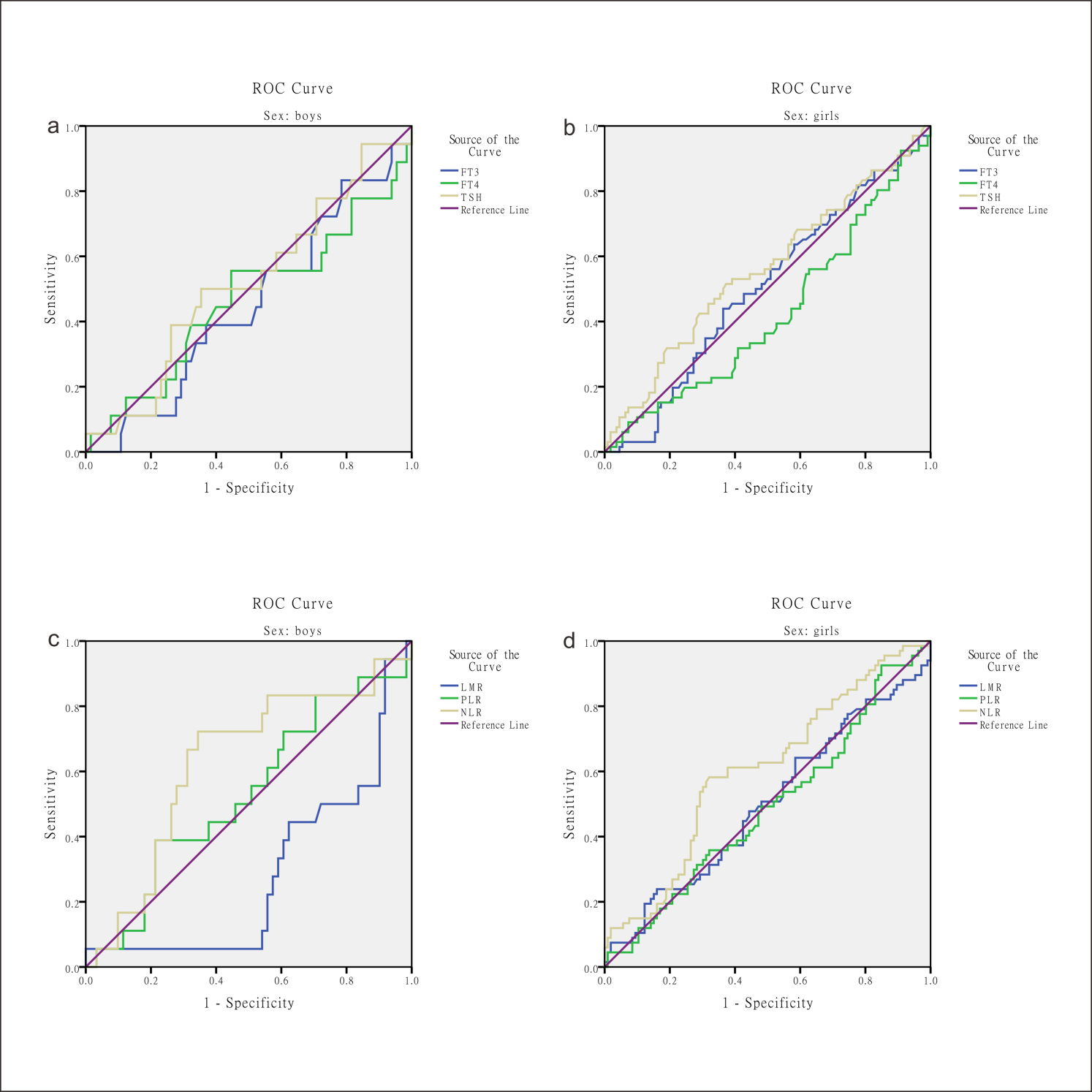
**

**Supplementary Figure 2.** ROC for determination of predictive ability of thyroid function and peripheral blood inflammatory indicators in pediatric thyroid cancer :a, thyroid function in boys group. b, thyroid function in girls group. c, peripheral blood inflammatory indicators in boys group. d, peripheral blood inflammatory indicators in girls group.

## Supplementary Tables

Supplementary Table1. Thyroid function and peripheral blood inflammatory indicators measurements by gender and age

|  |  | Age group | | | | | | |  | Gender group | | | | | | |
| --- | --- | --- | --- | --- | --- | --- | --- | --- | --- | --- | --- | --- | --- | --- | --- | --- |
|  |  | <14 | | |  | 14~17 | | |  | boys | | |  | girls | | |
|  |  | TC | TN | p |  | TC | TN | p |  | TC | TN | p |  | T | TN | p |
| FT3(pmol/L) |  | 5.93 | 5.85 | 0.967 |  | 5.245 | 0.017 | 0.827 |  | 5.595 | 5.68 | 0.507 |  | 5.45 | 5.4 | 0.984 |
| FT4(pmol/L) |  | 11.35 | 11.295 | 0.763 |  | 10.875 | 11.35 | 0.110 |  | 11.32 | 10.94 | 0.662 |  | 10.92 | 11.41 | 0.101 |
| TSH(μmIU/ML) |  | 3.359 | 2.54 | 0.201 |  | 2.47 | 2.075 | 0.306 |  | 2.29 | 2.14 | 0.821 |  | 3.24 | 2.51 | 0.193 |
| TPOAb |  | 8 | 9 | 0.076 |  | 10 | 9 | 0.082 |  | 1 | 3 | - |  | 17 | 15 | 0.041 |
| TGAb |  | 9 | 6 | 0.004 |  | 19 | 12 | 0.001 |  | 0 | 1 | - |  | 28 | 17 | 0.000 |
| LMR |  | 5.7 | 5.81 | 0.132 |  | 5.3 | 5.42 | 0.406 |  | 4.39 | 5.88 | 0.005 |  | 5.49 | 5.45 | 0.983 |
| NLR |  | 1.5 | 1.24 | 0.338 |  | 1.68 | 1.34 | 0.01 |  | 1.63 | 1.26 | 0.099 |  | 1.58 | 1.31 | 0.024 |
| PLR |  | 110.12 | 115.6 | 0.55 |  | 121.58 | 114.16 | 0.648 |  | 109.73 | 109.26 | 0.708 |  | 118.28 | 119.68 | 0.809 |

The significance level was set at p<0.05

Supplementary Table2. AUC of peripheral blood inflammatory indicators in pediatric thyroid cancer mersured by gender and age

|  |  | Age group | | | | | | |  | Gender group | | | | | | |
| --- | --- | --- | --- | --- | --- | --- | --- | --- | --- | --- | --- | --- | --- | --- | --- | --- |
|  |  | <14 | | |  | 14~17 | | |  | boys | | |  | girls | | |
|  |  | AUC | p | cut-off value |  | AUC | p | cut-off value |  | AUC | p | cut-off value |  | AUC | p | cut-off value |
| FT3 |  | 0.49 | 0.877 |  |  | 0.498 | 0.971 |  |  | 0.449 | 0.507 |  |  | 0.502 | 0.963 |  |
| FT4 |  | 0.487 | 0.848 |  |  | 0.418 | 0.082 |  |  | 0.466 | 0.662 |  |  | 0.424 | 0.093 |  |
| TSH |  | 0.585 | 0.201 |  |  | 0.549 | 0.306 |  |  | 0.518 | 0.821 |  |  | 0.559 | 0.193 |  |
| LMR |  | 0.401 | 0.132 |  |  | 0.460 | 0.406 |  |  | 0.281 | 0.005 |  |  | 0.499 | 0.983 |  |
| NLR |  | 0.563 | 0.338 |  |  | 0.623 | 0.01 | 1.50184 |  | 0.629 | 0.099 |  |  | 0.602 | 0.024 | 1.50023 |
| PLR |  | 0.461 | 0.550 |  |  | 0.522 | 0.648 |  |  | 0.529 | 0.708 |  |  | 0.489 | 0.809 |  |

The significance level was set at p<0.05
